# Effect of Seasonal Grazing on Ground-Dwelling Insect Communities in the Desert Steppe of Ningxia

**DOI:** 10.3390/insects16090939

**Published:** 2025-09-06

**Authors:** Chun Shi, Changyu Xiong, Ziyu Cao, Haixiang Zhang, Ying Wang, Wei Sun, Yifan Cui, Rong Zhang, Shuhua Wei

**Affiliations:** 1 College of Biological Science & Engineering, North Minzu University, Yinchuan 750021, China; sc450776071@163.com (C.S.); 15950550587@163.com (C.X.); czy200218@163.com (Z.C.); 2 Institute of Plant Protection, Ningxia Academy of Agriculture and Forestry Sciences, Yinchuan 750002, China; haixiangzhang@cau.edu.cn (H.Z.); wangying108@163.com (Y.W.); swlymyy@163.com (W.S.); sy20233243683@cau.edu.cn (Y.C.); yczhrnx@163.com (R.Z.); 3 College of Grassland Science and Technology, China Agricultural University, Beijing 100193, China

**Keywords:** seasonal grazing, desert steppe, ground-dwelling insect communities, insect diversity, regulatory mechanisms

## Abstract

To investigate the effects of seasonal grazing on ground-dwelling insect communities in desert steppe, this study conducted a controlled experiment in the desert steppe of Yanchi County, Ningxia, during 2022–2023. Five grazing regimes were established: spring-summer grazing (Sp+Su), spring-autumn grazing (Sp+Au), summer-autumn grazing (Su+Au), year-round continuous grazing (Annual), and no grazing (Control, CK). Insects were collected using pitfall traps and categorized into herbivorous and predatory functional groups. Combined with monitoring of vegetation community structure, we analyzed the regulatory mechanisms of grazing on insect diversity. The results showed that different grazing regimes had significantly divergent effects on herbivorous and predatory insects. Herbivorous insect diversity was significantly highest under the Annual grazing regime, while Sp+Au grazing effectively controlled herbivorous insect occurrence, resulting in the lowest abundance. Predatory insects exhibited the highest abundance but the lowest diversity under Su+Au grazing, whereas the CK regime increased their species richness. Beta diversity analysis indicated that total replacement diversity (Repl) was dominant, suggesting that grazing primarily influenced community structure by altering species composition rather than changing species number. Non-metric multidimensional scaling (NMDS) results revealed clustering characteristics of insect community structures under different grazing regimes. Redundancy analysis (RDA) and generalized additive models (GAMs) identified vegetation height and predatory insect abundance as key factors driving changes in herbivorous insects. Vegetation density and biomass exhibited nonlinear regulatory effects on herbivorous insects. Based on these findings, we recommend adopting either a hybrid strategy of “year-round continuous grazing combined with seasonal rest” or specifically the “spring + autumn” (Sp+Au) grazing regime. These approaches aim to synergistically achieve the goals of pest control and biodiversity conservation in desert steppe ecosystems.

## Simple Summary

This study conducted seasonal grazing experiments in the desert steppe of Yanchi, Ningxia, employing five grazing regimes: spring-summer (Sp+Su), spring-autumn (Sp+Au), summer-autumn (Su+Au), year-round (Annual), and no grazing (CK). The research aimed to unravel grazing’s regulatory mechanisms on surface-dwelling insect communities. Insects were collected via pitfall traps and categorized into phytophagous and predatory functional groups, coupled with vegetation monitoring. Key findings reveal: year-round grazing significantly enhances phytophagous insect diversity, while spring-autumn grazing effectively suppresses their abundance; summer-autumn grazing boosts predatory insect abundance but reduces their diversity, whereas no grazing elevates predatory species richness. Beta diversity analysis indicates grazing primarily restructures communities through species replacement (Repl dominance), not richness changes, with NMDS confirming distinct insect assemblages under each regime. RDA and GAM models identify vegetation height and predatory insect abundance as critical drivers of phytophagous insect dynamics, with vegetation density and biomass exerting nonlinear regulatory effects. The study proposes an integrated “year-round grazing + seasonal rest” or “spring-autumn grazing” strategy to synergize pest control and biodiversity conservation in desert grasslands.

## 1. Introduction

As a crucial agro-pastoral ecotone in northwestern China, Ningxia possesses abundant natural grassland resources. Among these, desert steppe constitutes the largest natural grassland type in the region, accounting for 59.06% of its total grassland area, and serves as a significant ecological barrier in China [1,2,3]. Grazing, as a primary utilization practice in grassland ecosystems, significantly influences landscape patterns by altering vegetation structure and community composition [4,5]. However, given the fragile ecological environment of desert steppes, long-term overgrazing can readily trigger vegetation degradation, soil erosion, and biodiversity loss, thereby threatening ecosystem stability and the sustainable development of animal husbandry [6]. Studies indicate that moderate grazing and seasonal rotational grazing can progressively restore grassland biodiversity and enhance primary productivity by maintaining vegetation heterogeneity and resource availability [7]. While grazing directly affects vegetation structure and productivity, it also indirectly shapes the structure and function of insect communities through modifications to plant community composition, microclimate conditions, and soil physicochemical properties [8]. Nevertheless, the mechanisms underlying grazing impacts on insect communities are complex, and the direction of these effects (promotive or suppressive) varies depending on insect taxa, grazing intensity, and vegetation type [9].

As the most diverse animal taxa, insects constitute a vital component of grassland ecosystems [10]. Their extensive habitat range and sensitivity to environmental changes make them valuable indicator species for assessing grassland ecosystem health [11,12,13,14]. Grazing influences insect communities through direct pathways (livestock trampling and accidental ingestion) and indirect pathways (alterations to vegetation structure, microclimate, and food resources) [15,16,17,18]. For instance, high-intensity grazing significantly reduces vegetation cover and height, leading to increased ground surface temperature and decreased humidity, which consequently diminishes the abundance of sciophilous (shade-preferring) insects [19]. Conversely, patchy vegetation generated by moderate grazing can provide habitats for insects occupying different ecological niches, thereby enhancing community diversity [8]. Notably, grazing effects exhibit taxon-specificity: Orthopteran abundance increases under light grazing due to greater availability of tender plant tissues [15,20], whereas predatory insects such as carabid beetles show higher diversity in rotational grazing areas owing to reduced soil disturbance [21]. The impacts of different grazing regimes on insects are significant. Compared to single-species grazing, mixed livestock grazing (co-grazing by cattle and sheep) enhances vegetation heterogeneity through differential foraging strategies, proving more beneficial for maintaining insect diversity [15,22]. Furthermore, seasonal variations in grazing exert distinct effects on insect diversity. Spring grazing exerts stronger suppressive effects on Orthoptera than autumn grazing, attributable to differences in their developmental stages [20,23].

The desert steppe of Ningxia, located in an arid zone, experiences scarce precipitation and intense evaporation, rendering its ecosystem particularly sensitive to grazing disturbances [24]. Although previous studies have provided some insights into Ningxia’s desert steppe and its insect communities, several critical knowledge gaps remain. For instance, long-term monitoring data documenting insect community dynamics under different grazing regimes are still relatively scarce, hindering accurate assessment of temporal trends [25,26,27]. Concurrently, the complex interrelationships between changes in insect community structure and other biotic and abiotic factors within the grassland ecosystem lack systematic investigation. Notably, the sensitivity of soil insect communities to environmental changes has been well-documented [27,28,29], a characteristic that makes them excellent bio-indicators for assessing grassland ecological health. Within this contex, building upon the framework of seasonal grazing, this study investigates the impact of seasonally-crossed grazing regimes on insect communities in the Ningxia desert steppe. By comparing the dynamic changes in insect community composition and vegetation community structure indices across these different regimes, we elucidate the regulatory roles of distinct grazing patterns on the responses of herbivorous insect diversity to predatory insect diversity indices and vegetation community structural characteristics. This research not only advances the understanding of desert steppe ecosystems but also provides a theoretical foundation for developing integrated grazing management strategies that synergize pest control and biodiversity conservation in these fragile landscapes.

## 2. Materials and Methods

### 2.1. Overview of the Research Area

**Figure 1:**
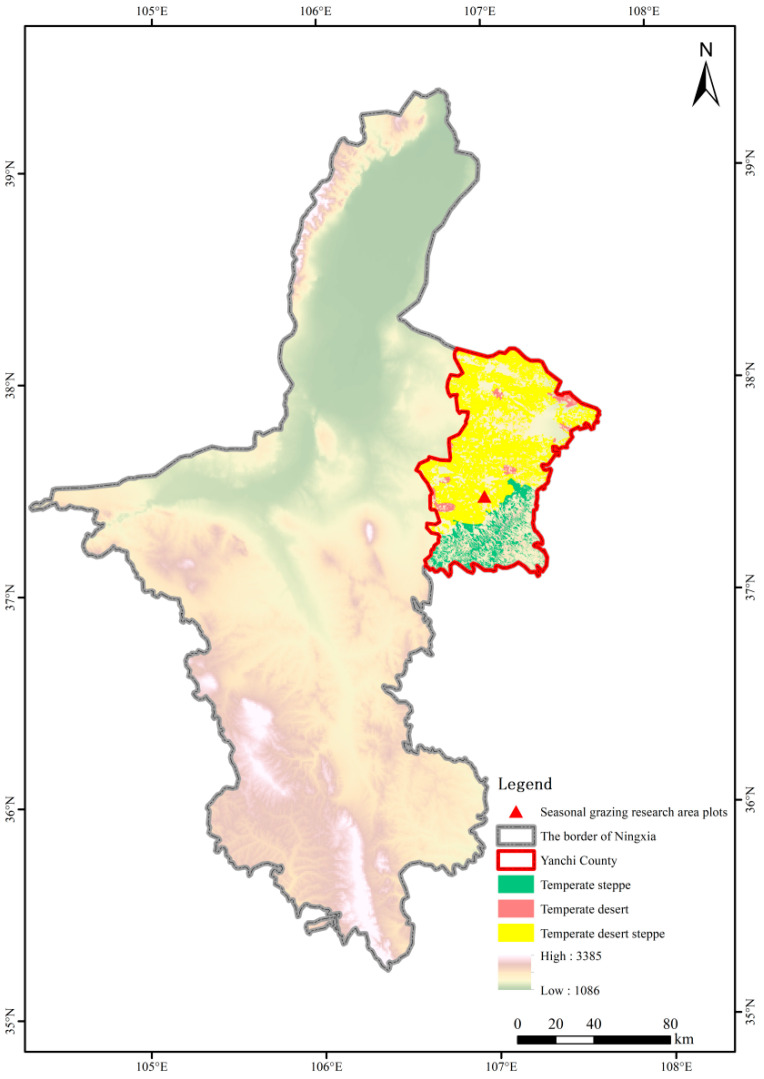
Geographic location of the seasonal grazing experimental site.

The study site is located in Yanchi County (37°04′–38°10′ N, 106°30′–107°47′ E), eastern Ningxia, situated at the junction of Shaanxi, Gansu, Ningxia, and Inner Mongolia provinces. Elevation ranges between 1295 and 1951 m, positioning it within China’s agro-pastoral ecotone. The local climate is characterized as a typical temperate continental climate, with an average annual frost-free period of 188 days, mean annual temperature of 7.8 °C, and annual precipitation of 250–350 mm [30]. Within this area, the grazing experimental plots were established in 2019 at Dashuikeng Town (37°24′19″ N, 106°58′48″ E). Prior to establishment, the site consisted of semi-natural temperate desert steppe (Figure 1).

### 2.2. Method

#### 2.2.1. Experiment Design

**Figure 2:**
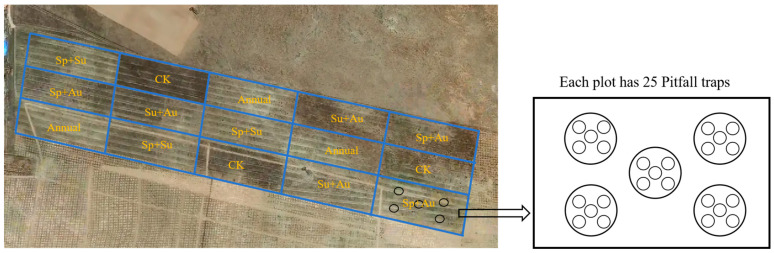
Layout of experimental plots under seasonal grazing regimes. Note: Sp+Su denotes spring-summer grazing, Sp+Au spring-autumn grazing, Su+Au summer-autumn grazing, Annual year-round continuous grazing, and CK no-grazing control.

The experimental design employed a controlled trial to systematically investigate the mechanisms by which seasonal grazing affects ground-dwelling insect communities in Ningxia’s desert steppe. Five distinct grazing treatments were implemented: spring-summer grazing (Sp+Su), spring-autumn grazing (Sp+Au), summer-autumn grazing (+Au), year-round continuous grazing (Annual), and a no-grazing control (CK). Each treatment was replicated three times, resulting in a total of fifteen experimental plots. Each grazing enclosure covered an area of 9000 m^2^ (180 m × 50 m). Adjacent plots were physically isolated by 1.2-m-high fencing to prevent cross-interference (Figure 2). Based on aboveground net primary productivity (ANPP) measurements from reseeded grasslands during preliminary studies (2019–2020) and the optimal stocking rate for natural steppe, a moderate grazing intensity of four adult sheep (35 ± 1.2 kg) per plot was established. The grazing period spanned 2022 to 2023. Systematic surveys of ground-dwelling insect communities were conducted: (1) pre-grazing, and (2) during spring, summer, and autumn seasons post-grazing implementation. This longitudinal monitoring captured changes in steppe insect communities across grazing regimes, enabling analysis of grazing impacts on insect assemblages.

#### 2.2.2. Collection of Insects

Ground-dwelling insects were sampled using pitfall traps. The trapping protocol was as follows: Within each experimental plot, five sampling points were established in a five-point sampling pattern. At each point, five traps were deployed with 5-m intervals between adjacent traps. This resulted in 25 traps per plot, totaling 375 traps across all 15 plots. Disposable transparent cups (height: 11.5 cm; diameter: 9 cm) were buried with their rims flush with the ground surface. Each cup was filled to one-third of its volume with trapping solution (ethylene glycol:water = 1:2, *v*/*v*, ethylene glycol was purchased from Tianjin Fuyu Fine Chemical Co., Ltd., Tianjin, China). Insects were collected at 10-day intervals and preserved in 95% ethanol, ethanol was purchased from Tianjin Beilian Fine Chemicals Development Co., Ltd., Tianjin, China [1]. All insect specimens were transported to the laboratory for pinning and mounting. Species identification and documentation were conducted primarily with reference to the following literature: *Colored Pictorial Handbook of Grassland Insects in Ningxia* [31], *Fauna of the Beetles from Ningxia, China* [32], and *Insects of Helan Mountain in Ningxia* [33]. Insects were classified into herbivorous and predatory groups (see Tables S1 and S2 for details) for analysis based on a comprehensive examination of individual morphological characteristics (e.g., mouthpart types, appendage structures) and documented ecological records of the taxonomic units (as referenced in literature sources [31,32,33]). The determination of dominant species was based on the proportion of individual numbers of each insect species to the total number of individuals in the community. Species with a proportion below 1% were classified as rare species, those between 1% and 10% as common species, and those exceeding 10% as dominant species [34].

#### 2.2.3. Vegetation Investigation

Within each grazing plot, three permanent 1 m × 1 m quadrats were randomly established where plant species abundance was recorded by counting all individuals per species; additionally, five randomly selected plants per species were measured for height using a measuring tape. Each quadrat was subdivided into a grid of 100 contiguous 10 cm × 10 cm subquadrats, and vegetation cover was quantified using the point-quadrat method: at each corner of every subquadrat (four points per subquadrat), a vertical needle was lowered to record plant species contacts and repeat hits (contacting two species counted as one repeat hit). Total vegetation cover was calculated as the sum of species-specific cover values minus repeat hits [35]. Following cover, density, and height measurements, all aboveground vegetation within quadrats was cut at ground level, sorted by species into envelopes, dried at 80 °C for 24 h in an electric thermostatic drying oven, electric thermostatic was purchased from Ningbo Jiangnan Instrument Works, Ningbo, China and weighed to 0.001 g precision using an electronic balance to determine community aboveground biomass, electronic balance was purchased from Changshu Tianliang Instrument Co., Ltd., Changshu, China.

#### 2.2.4. Calculation of the Diversity Index

Beyond species richness and abundance, insect diversity was assessed using four indices: The Margalef richness index (d) primarily evaluates and compares species diversity levels across ecological communities by quantifying the relationship between species number and total individuals, reflecting community richness characteristics while being insensitive to species evenness; the Shannon-Wiener diversity index (H’) incorporates both species richness and evenness while being sensitive to the presence of rare species. It quantifies community heterogeneity through species composition and relative abundance, and is often used as an indicator of ecosystem stability; the Simpson dominance index (λ) quantifies the proportional abundance of the most dominant species, with higher values indicating lower overall diversity due to dominance by few species; and the Pielou evenness index (E), derived from the Shannon-Wiener index, specifically measures the equitability of species abundance distribution, where E = 1 when all species are equally abundant, indicating maximal community equilibrium [15,36].

Non-metric Multidimensional Scaling (NMDS) based on Bray–Curtis dissimilarity was applied to visualize differences in insect community structure across grazing regimes. This method effectively handles the non-linear nature of ecological data and projects ecological distances between samples into a low-dimensional ordination space. Stress values were used to evaluate the reliability of the NMDS configuration; a value below 0.2 generally indicates good interpretability. To further investigate the disparities in community structure under different grazing conditions and their underlying drivers, beta diversity was partitioned into two ecologically meaningful components: species turnover (Repl) and richness differences (Rich) [2].

Generalized Additive Models (GAMs) were constructed to quantify the linear/non-linear relationships between herbivorous insect abundance (HI_Abundance) (response variable, continuous) and environmental predictors (explanatory variables). Explanatory variables were selected based on prior Redundancy Analysis (RDA) and Mantel tests, which identified key drivers of herbivorous insect communities.

### 2.3. Data Analysis

Experimental data were organized using Microsoft Excel 2021 (Microsoft Corporation, Redmond, WA, USA). Diversity composition differences in insect communities across grazing regimes were estimated via the “iNEXT” package (v.4.0.0) in R 4.4.1(R Core Team, Vienna, Austria) [37]. Differences in insect diversity and vegetation structure among grazing treatments were tested using one-way ANOVA with Duncan’s multiple range test. NMDS ordination was implemented with the metaMDS function (“vegan” v.2.6-4) [38], while beta diversity partitioning was performed using beta.div.comp (“adespatial” v.0.3-21) [39]. Detrended correspondence analysis (DCA) for dominant carabid species and diversity was conducted via the decorana function (“vegan”). Based on DCA axis lengths (thresholds: RDA if <3.0, CCA if <4.0) [40], either redundancy analysis (RDA) or canonical correspondence analysis (CCA) was selected for examining insect-vegetation relationships using rda/cca functions (“vegan”). Mantel tests (“LinkET” v.0.0.3) calculated Bray-Curtis and Euclidean distances between species, followed by Mantel correlations between herbivorous insects and environmental factors, supplemented by Pearson correlation analysis among environmental variables [41]. Generalized additive models (GAMs) were constructed using the “mgcv” package (v.1.9-0) [42], employing non-parametric smoothing terms to capture linear/non-linear relationships between herbivorous insects and environmental predictors. All visualizations were generated with “ggplot2” (v.3.4.4) and GraphPad Prism 9.5.0 (GraphPad Software, San Diego, CA, USA).

## 3. Result and Analysis

### 3.1. Insect Distribution in Grazing Areas

**Figure 3:**
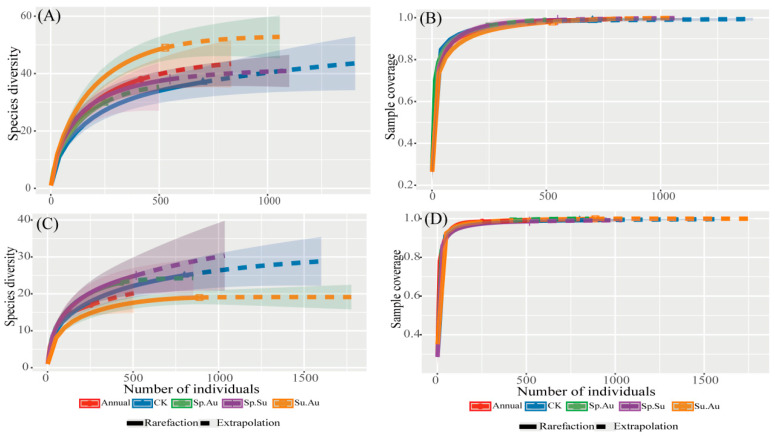
Insect community diversity across grazing regimes based on rarefaction extrapolation analysis. Note: (**A**) Rarefaction/extrapolation curves for herbivorous insect samples. (**B**) Sample completeness curve for herbivorous insects. (**C**) Rarefaction/extrapolation curves for predatory insect samples. (**D**) Sample completeness curve for predatory insects. Solid lines were generated by interpolation, dashed lines by extrapolation.

Based on the rarefaction-extrapolation curves of insect samples under different grazing regimes (Figure 3), the curves in Figure 3A,C demonstrate the asymptotic stabilization of diversity with increasing sample size. Figure 3B,D display the sample coverage curves, whose rising trajectories indicate continuous improvement in the coverage of total species richness with additional sampling, all exceeding 99% and asymptotically approaching a value of 1. This indicates that each grazing regime achieves sufficient coverage of species diversity at the current sample size. These results fully demonstrate that the sampling effort meets the required standards and that the data obtained are reliable for assessing community diversity.

### 3.2. Insect Diversity in Grazing Areas

#### 3.2.1. Alpha Diversity Analysis of Herbivorous Insects and Predatory Insects

**Figure 4:**
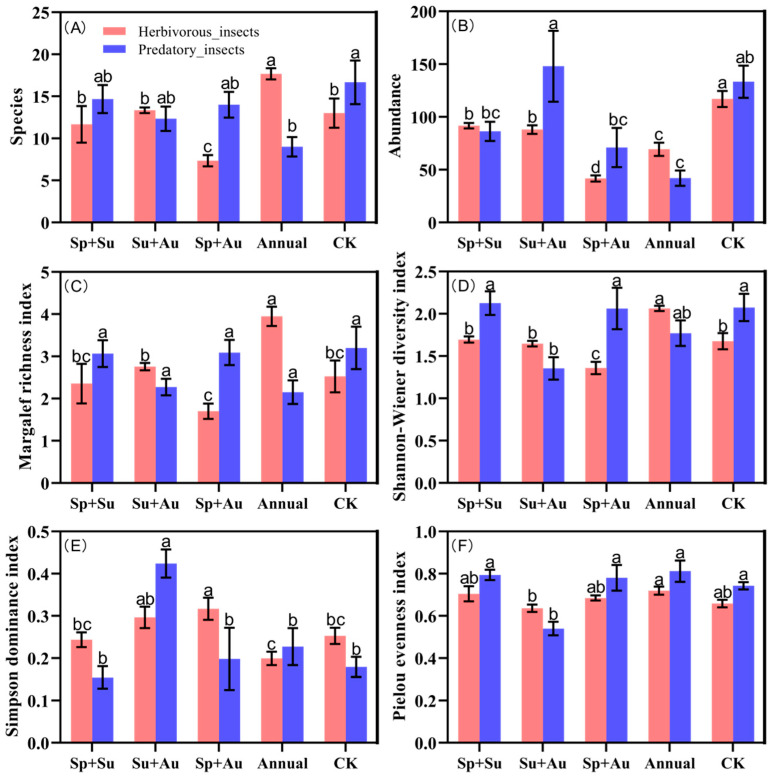
Alpha diversity of insect communities across grazing regimes. Note: Red bars denote herbivorous insects, blue bars predatory insects. Data represent mean ± SE. Based on Duncan’s new multiple range test (DMRT), different lowercase letters indicate significant differences in diversity indices across grazing regimes (*p* < 0.05), while identical letters denote non-significant differences. (**A**) The number of herbivorous and predatory insect species collected under different grazing regimes. (**B**) The abundance of herbivorous and predatory insects collected under different grazing regimes. (**C**) Margalef richness index of herbivorous and predatory insects collected under different grazing regimes. (**D**) Shannon-Wiener diversity index of herbivorous and predatory insects collected under different grazing regimes. (**E**) Simpson dominance index of herbivorous and predatory insects collected under different grazing regimes. (**F**) Pielou evenness index of herbivorous and predatory insects collected under different grazing regimes.

Significant variations emerged among herbivorous insect diversity indices across grazing regimes (red bars, Figure 4). For species richness: Year-round grazing (Annual) yielded the highest richness, significantly exceeding other regimes (*p* < 0.05). Spring-autumn grazing (Sp+Au) exhibited the lowest richness, while spring-summer (Sp+Su), summer-autumn (Su+Au), and no-grazing (CK) showed statistically comparable richness levels (*p* > 0.05), indicating similar regulatory effects on herbivore richness among these three treatments (Figure 4A). Regarding abundance: CK demonstrated the highest herbivore abundance, significantly surpassing grazed treatments (*p* < 0.05). Sp+Su and Su+Au maintained intermediate abundance levels, whereas Sp+Au resulted in minimal abundance (Figure 4B). Margalef and Shannon-Wiener indices both peaked under Annual grazing, showing significant advantages over other regimes (*p* < 0.05), while reaching their minima under Sp+Au. This reflects weakened species accumulation capacity and significantly reduced diversity under Sp+Au. Intermediate values without significant differences characterized the remaining treatments (Figure 4C,D). Simpson dominance index was elevated under Su+Au and Sp+Au but minimized under Annual grazing (Figure 4E). Pielou evenness exhibited modest differences across regimes, indicating relatively balanced distribution of herbivorous individuals within communities (Figure 4F).

Predatory insect community diversity exhibited distinct patterns from herbivorous insects (blue bars, Figure 4). Species richness peaked under no-grazing (CK), significantly exceeding year-round grazing (Annual) (*p* < 0.05), with no significant differences among the other three regimes (Figure 4A). For abundance: Summer-autumn grazing (Su+Au) yielded the highest predatory insect abundance, significantly surpassing spring-summer (Sp+Su), spring-autumn (Sp+Au), and Annual regimes (*p* < 0.05). Abundance levels remained statistically comparable among Sp+Su, Su+Au, and CK (Figure 4B). Margalef richness index showed no significant differences across regimes (Figure 4C). The Shannon-Wiener index was significantly depressed under Su+Au compared to Sp+Su, Sp+Au, and CK (*p* < 0.05), while maintaining comparable values among the latter four regimes (Figure 4D). Simpson dominance peaked under Su+Au, significantly exceeding all other treatments (*p* < 0.05) (Figure 4E). Conversely, Pielou evenness reached its minimum under Su+Au, significantly lower than other regimes (*p* < 0.05) (Figure 4F).

#### 3.2.2. Beta Diversity Analysis of Herbivorous Insects and Predatory Insects

**Figure 5:**
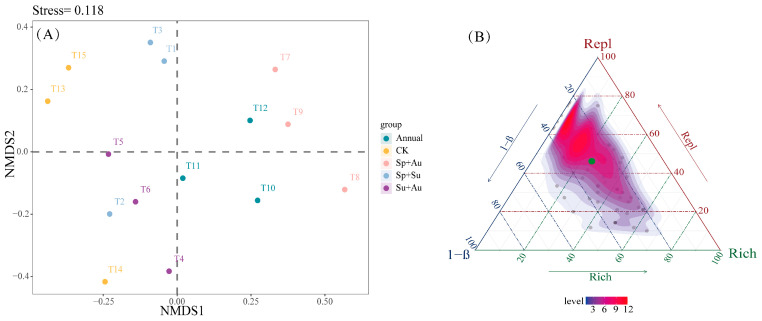
NMDS ordination and beta diversity partitioning of herbivorous insects across grazing regimes. Note: (**A**) Compositional differences of herbivorous insect communities across grazing regimes, where points represent individual experimental quadrats. (**B**) Ternary plot depicting total beta diversity and its components (Repl: replacement diversity; Rich: richness difference) under different regimes. Green dots indicate mean beta diversity values (hereafter identical).

Non-metric multidimensional scaling (NMDS) analysis of herbivorous insect communities (Stress = 0.118) indicated high reliability in ordination outcomes, as the stress value below 0.2 effectively captured structural differences among samples. Specifically, Year-round (Annual) and spring-autumn (Sp+Au) grazing samples formed proximal clusters in ordination space, reflecting similar community structures, while no-grazing (CK), spring-summer (Sp+Su), and summer-autumn (Su+Au) treatments exhibited adjacent clustering patterns, indicating structural similarity among these three regimes (Figure 5A). Beta diversity partitioning revealed a total β diversity of 0.3465 for herbivorous insects, with mean replacement diversity (Repl) at 0.2308 and mean richness difference diversity (Rich) at 0.1224, demonstrating Repl’s dominant contribution (65.35%) to overall beta diversity (Figure 5B).

**Figure 6:**
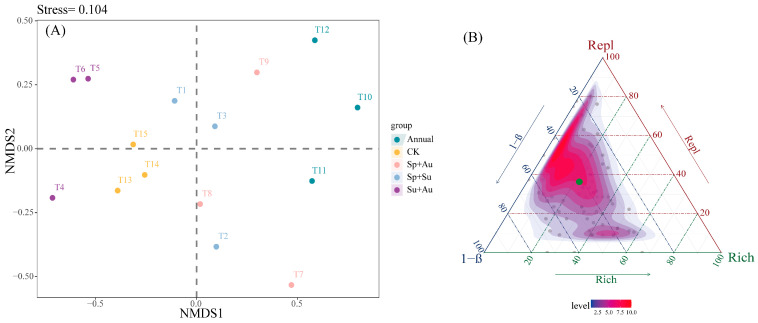
NMDS ordination and beta diversity partitioning of predatory insects across grazing regimes. Note: (**A**) Compositional differences of predatory insect communities across grazing regimes, where points represent individual experimental quadrats. (**B**) Ternary plot depicting total predatory insect beta diversity and its components (Repl: replacement diversity; Rich: richness difference) under different regimes. Green dots indicate mean beta diversity values (hereafter identical).

Non-metric multidimensional scaling (NMDS) analysis of predatory insect communities (Stress = 0.104) confirmed high ordination reliability, as the sub-0.2 stress value effectively captured structural differences among samples. Specifically: Year-round grazing (Annual) samples clustered in the upper-middle sector of NMDS2 axis, showing clear spatial separation from other regimes; spring-autumn (Sp+Au) and spring-summer (Sp+Su) grazing samples occupied adjacent ordination spaces, indicating structural similarity; summer-autumn (Su+Au) samples dispersed across the lower-middle NMDS1 and lower-middle NMDS2 quadrants with minimal overlap; while no-grazing (CK) samples positioned in the lower-central region exhibited partial spatial overlap with multiple treatments, reflecting both connections and distinctions in community structure (Figure 6A). Beta diversity partitioning revealed total β diversity of 0.2917, with mean replacement (Repl) and richness difference (Rich) components at 0.1816 and 0.1101 respectively, demonstrating Repl’s dominant contribution (62.27%) to overall beta diversity (Figure 6B).

### 3.3. Vegetation Community Structure Index of Grazing Areas

**Figure 7:**
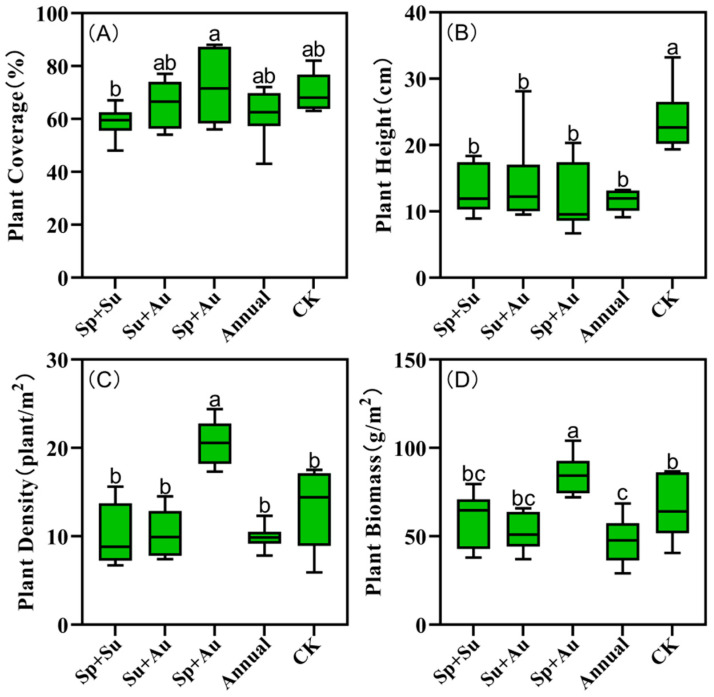
Vegetation structural indices in grazing plots. Note: Data represent mean ± SE. Based on Duncan’s new multiple range test (DMRT), different lowercase letters indicate significant differences in vegetation structural indices across grazing regimes (*p* < 0.05), while identical letters denote non-significant differences. (**A**) Plant cover of plots under different grazing regimes. (**B**) Plant height of plots under different grazing regimes. (**C**) Plant density of plots under different grazing regimes. (**D**) Plant biomass of plots under different grazing regimes.

Vegetation structural indices varied across grazing regimes, with spring-autumn grazing (Sp+Au) exhibiting peak values in coverage (72.17%), density (20.58 plants/m^2^), and aboveground biomass (84.78 g/m^2^) (Figure 7A,C,D). Notably, Sp+Au significantly exceeded other regimes in density and biomass (*p* < 0.05), while its coverage was significantly higher than spring-summer grazing (Sp+Su) (*p* < 0.05). Conversely, no-grazing (CK) yielded maximum vegetation height (23.77 cm), significantly surpassing all grazed treatments (*p* < 0.05) (Figure 7B).

### 3.4. The Relationship Between Herbivorous Insects and Environmental Factors

#### 3.4.1. Redundancy Analysis of Herbivorous Insects and Environmental Factors

**Figure 8:**
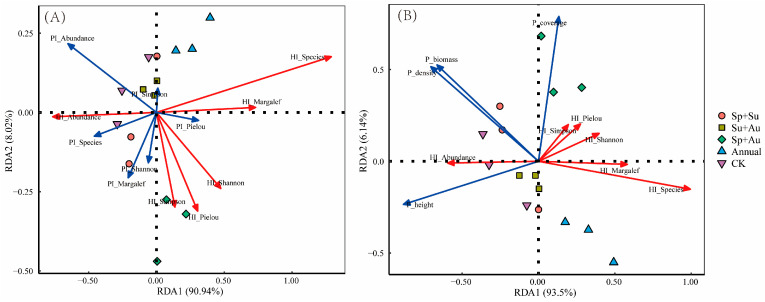
RDA ordination of herbivorous insect diversity in response to environmental predictors across grazing regimes. Note: (**A**) RDA ordination between herbivorous and predatory insect diversity indices. (**B**) RDA ordination between herbivorous insect diversity and vegetation structural parameters. Abbreviations: HI_Species: Herbivorous insect species richness; HI_Abundance: Herbivorous insect abundance; HI_Margalef: Margalef richness index of herbivores; HI_Shannon: Shannon-Wiener diversity index of herbivores; HI_Simpson: Simpson dominance index of herbivores; HI_Pielou: Pielou evenness index of herbivores; P_coverage: Vegetation coverage; P_height: Vegetation height; P_density: Vegetation density; P_biomass: Aboveground biomass (hereafter identical). Red arrows represent herbivorous insect diversity indices; blue arrows denote environmental factors. Acute angles between arrows indicate positive correlations; obtuse angles indicate negative correlations.

Redundancy analysis (RDA) of herbivorous (HI) and predatory insects (PI) diversity indices demonstrated that the first two axes cumulatively explained 98.96% of variation (RDA1: 90.94%; RDA2: 8.02%), with PI indices influencing herbivorous insects in descending order: PI_Abundance > PI_Margalef > PI_Species > PI_Shannon > PI_Pielou > PI_Simpson. HI_Abundance showed positive correlations with PI_Abundance, PI_Species, PI_Margalef, and PI_Shannon; conversely, HI_Species and HI_Margalef exhibited negative correlations with these same PI indices, while HI_Shannon, HI_Pielou, and HI_Simpson were negatively correlated specifically with PI_Margalef and PI_Shannon (Figure 8A).

Redundancy analysis (RDA) examining relationships between herbivorous insect (HI) diversity indices and vegetation structural parameters revealed that the first two RDA axes cumulatively explained 99.64% of variation (RDA1: 93.5%; RDA2: 6.14%), with vegetation indices influencing herbivorous insects in descending order: P_coverage > P_height > P_density > P_biomass. HI_Abundance showed positive correlations with P_height, P_density, and P_biomass but a negative correlation with P_coverage; conversely, HI_Species, HI_Margalef, HI_Shannon, HI_Pielou, and HI_Simpson all exhibited negative correlations with P_height, P_density, and P_biomass (Figure 8B).

#### 3.4.2. Mantel Analysis of Herbivorous Insects and Environmental Factors

**Figure 9:**
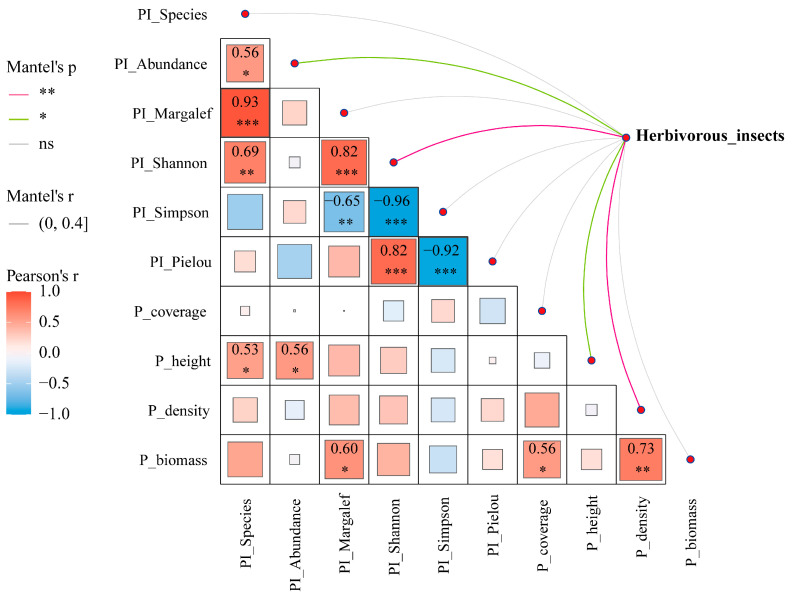
Mantel correlogram of environmental factors and their correlations with herbivorous insect communities. Note: *** denotes *p* < 0.001, ** denotes *p* < 0.01, * denotes *p* < 0.05 and ns denotes no significance.

Mantel test-based ecological association analysis revealed significant correlations between herbivorous insect communities and predatory insect diversity indices/vegetation structural parameters across grazing regimes. Results demonstrated that PI_Abundance and P_height exerted significant positive effects on herbivorous insect communities (*p* < 0.05), while PI_Shannon and P_height showed stronger positive driving effects (*p* < 0.01). Additionally, P_height exhibited significant positive relationships with PI_Species and PI_Abundance (*p* < 0.05), and P_biomass was positively correlated with PI_Margalef (*p* < 0.05) (Figure 9).

#### 3.4.3. Responses of Herbivorous Insects to the Diversity of Predatory Insects

**Figure 10:**
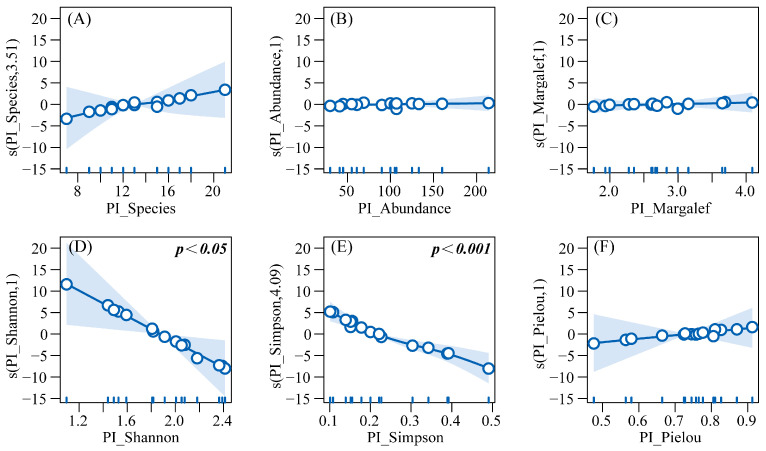
GAM response curves of herbivorous insect abundance to predatory insect diversity. Note: (**A**) Effect of predatory insect species richness on the abundance of herbivorous insects. (**B**) Effect of predatory insect abundance on the abundance of herbivorous insects. (**C**) Effect of the Margalef richness index of predatory insects on the abundance of herbivorous insects. (**D**) Effect of the Shannon-Wiener diversity index of predatory insects on the abundance of herbivorous insects. (**E**) Effect of the Simpson dominance index of predatory insects on the abundance of herbivorous insects. (**F**) Effect of the Pielou evenness index of predatory insects on the abundance of herbivorous insects.

GAMs were built with HI_Abundance as the response variable and six predatory insect diversity indices (PI_Abundance, PI_Species, etc.) as explanatory variables, to explore their linear/non-linear associations. Results from the Generalized Additive Model (R^2^ = 0.945, Deviance explained = 98.6%) indicated that herbivorous insect abundance exhibited linear positive correlations with predatory insect abundance (PI_Abundance), Margalef richness index (PI_Margalef), and Pielou evenness index (PI_Pielou), though these correlations were not statistically significant. In contrast, a significant linear negative correlation was observed with the Shannon diversity index (PI_Shannon) (*p* < 0.05). Non-linear relationships were identified as follows: herbivorous insect abundance increased with predatory species richness (PI_Species), but the growth rate decelerated when species richness exceeded 12; meanwhile, abundance decreased significantly with increasing Simpson dominance index (PI_Simpson) (*p* < 0.001), with the decline rate moderating within the range of 0.2–0.4 and accelerating beyond 0.4 (Figure 10).

#### 3.4.4. The Response of Herbivorous Insects to the Structure Index of Vegetation Communities

**Figure 11:**
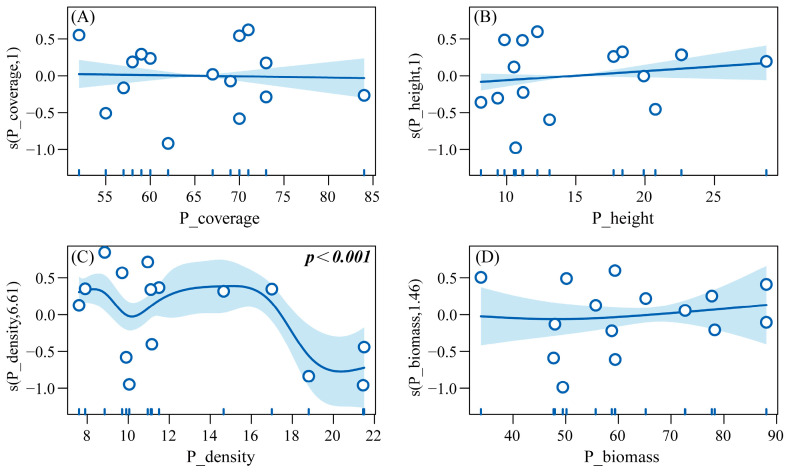
GAM response curves of herbivorous insect abundance to vegetation structural indices. Note: (**A**) Effect of plant cover on the abundance of herbivorous insects. (**B**) Effect of plant height on the abundance of herbivorous insects. (**C**) Effect of plant density on the abundance of herbivorous insects. (**D**) Effect of plant biomass on the abundance of herbivorous insects.

GAMs were built with HI_Abundance as the response variable and six predatory insect diversity indices (PI_Abundance, PI_Species, etc.) as explanatory variables, to explore their linear/non-linear associations. Results from the Generalized Additive Model (R^2^ = 0.927, Deviance explained = 97.9%) revealed that under different grazing regimes, herbivorous insect abundance exhibited the following relationships with vegetation parameters: a non-significant negative correlation with vegetation coverage (P_coverage) and a non-significant positive correlation with vegetation height (P_height). In contrast, a significant non-linear relationship was observed with vegetation density (P_density) (*p* < 0.001), while a non-significant non-linear correlation was found with biomass (P_biomass). Specifically, herbivorous insect abundance showed a negative correlation with vegetation density within the range of 8–10 plants/m^2^, transitioned to a positive correlation between 10–16 plants/m^2^, and reverted to a negative correlation at 16–20 plants/m^2^. Regarding biomass, a negative correlation was observed below the threshold of 50 g/m^2^, which shifted to positive above this value (Figure 11).

## 4. Discussion

This study systematically investigated the effects of seasonal grazing regimes on ground-dwelling insect communities in Yanchi County’s desert steppe of Ningxia. By comparing dynamic changes in insect communities and vegetation characteristics across five grazing treatments, we revealed differential impacts on herbivorous and predatory insect diversity, and elucidated how distinct grazing patterns modulate herbivorous insect responses to predatory insect diversity indices and vegetation structural parameters. These findings not only provide novel evidence for understanding the maintenance mechanisms of insect diversity in arid grazing ecosystems, but also establish a theoretical foundation for developing integrated management strategies that synergistically achieve pest control and biodiversity conservation objectives.

This study revealed significant differential effects of grazing regimes on herbivorous versus predatory insects. Year-round continuous grazing significantly enhanced herbivorous insect species richness and Shannon diversity while reducing their dominance, suggesting this regime maintains high vegetation heterogeneity that provides resources and habitats for niche-differentiated herbivores [43]. Conversely, predatory insects exhibited minimal richness and moderate-to-low abundance under year-round grazing, indicating sustained disturbance may suppress predators through soil compaction and reduced microhabitat complexity [44]. Summer-autumn grazing uniquely increased predatory insect abundance but significantly decreased Shannon diversity and evenness, promoting dominance by few species. This pattern implies that seasonal grazing pulses may concentrate predators via herbivore resource pulses, yet simultaneously destabilize communities [45]. No-grazing plots maximized predatory insect richness, likely due to complex refuge-hunting grounds provided by taller, denser vegetation for carabids and spiders [46]. However, elevated herbivore abundance here signals long-term grazing exclusion risks triggering pest outbreaks [47]. These findings confirm taxon-specific grazing effects modulated by seasonal dynamics, aligning with prior Ningxia steppe research [48].

Beta diversity decomposition revealed that replacement diversity (Repl) accounted for over 60% of total β-diversity in both herbivorous and predatory insect communities, indicating that seasonal grazing primarily influences community structure through species turnover rather than simple richness changes. This finding supports the Intermediate Disturbance Hypothesis [49], whereby moderate heterogeneous grazing creates diversified microhabitats that drive niche differentiation and species coexistence through differential species responses to environmental heterogeneity [50]. NMDS ordination further demonstrated clustering of year-round and spring-autumn grazing regimes in herbivorous insect structure, potentially attributable to their shared maintenance of relatively high proportions of perennial forbs that provide continuous food resources for generalist herbivores [51]. Conversely, summer-autumn grazing showed clear separation from no-grazing in predatory insect structure, likely filtering predatory communities through altered near-ground thermal-moisture conditions that drive functional group reassembly [52].

Redundancy analysis and Mantel tests identified vegetation height and predatory insect abundance as key drivers of herbivorous insect community dynamics. The positive correlation between herbivorous abundance and vegetation height likely reflects enhanced foraging resources and oviposition sites provided by taller vegetation [53], while the positive linkage with predatory abundance validates a top-down control effect—increased herbivores supply more prey, fostering synergistic growth [54]. GAM response curves revealed non-linear relationships between herbivorous abundance and vegetation density/biomass: positive correlations emerged at intermediate densities (10–16 plants/m^2^), but turned negative outside this range. This optimal window likely balances resource availability with minimized microhabitat deterioration from overcrowding, validating the Intermediate Disturbance Hypothesis in desert steppes [55]. When biomass exceeded 50 g/m^2^, herbivorous abundance surged significantly, indicating release from resource limitation beyond this productivity threshold [56]—a potential precursor to pest outbreaks. Conversely, vegetation coverage negatively correlated with herbivorous abundance, possibly by suppressing ground temperature rises critical for sun-preferring insects’ development in arid habitats [57]. These threshold-driven relationships underscore vegetation’s pivotal role in insect community regulation, providing quantitative benchmarks for precision grazing management in desert steppes. Strategically modulating vegetation density and biomass enables preemptive regulation of key herbivorous populations.

Diverging from single functional group studies, our findings reveal significant response disparities between herbivorous and predatory insects, necessitating balanced management strategies accommodating both groups. While spring-autumn grazing enhances vegetation productivity, it may compromise ecosystem multifunctionality by excessively reducing herbivorous insect diversity. Conversely, year-round grazing boosts herbivore diversity but risks triggering pest outbreaks due to predator suppression. This perspective aligns with the philosophy that different insect functional groups should be considered comprehensively in ecological research [48].

## 5. Conclusions

Conducted within Ningxia’s unique desert steppe ecosystem, this study proposes tailored grazing management strategies based on empirical findings: For biodiversity conservation objectives, a hybrid regime of “year-round grazing with seasonal rests” is recommended to maintain herbivorous insect diversity while preserving intact habitats for predators. To control herbivore overpopulation, spring-autumn grazing should be prioritized, leveraging its characteristic high vegetation biomass and low herbivore abundance to mitigate pest risks. Limitations warrant consideration: The two-year study period necessitates long-term monitoring to assess cumulative grazing effects on insect communities. Furthermore, impacts on soil microbiota and litter decomposition remain unexamined future research should incorporate multifactorial interactions to deepen mechanistic understanding. Additionally, differential effects of livestock species (e.g., mixed cattle and sheep grazing) represent a critical knowledge gap for refining management precision. In summary, this work elucidates how seasonal grazing modulates desert steppe ecosystems through vegetation and insect interactions, offering scientific support for sustainable utilization and biodiversity conservation in these fragile landscapes.

## Data Availability

The data presented in this study are available upon request from the corresponding author.

## Supplementary Materials

The following supporting information can be downloaded at: https://www.mdpi.com/article/10.3390/insects16090939/s1, Table S1: The species and abundance of herbivorous insects collected under different grazing regimes. Table S2: The species and abundance of predatory insects collected under different grazing regimes.

## Abbreviations

The following abbreviations are used in this manuscript:

Sp	Spring
Su	Summer
Au	Autumn
ANPP	Aboveground net primary productivity
HI	Herbivorous insects
PI	Predatory insects
P	Plants
