# Supplementary material for: Effect of Seasonal Grazing on Ground-Dwelling Insect Communities in the Desert Steppe of Ningxia
Source: Insects. 2025 Sep 6;16(9):939. doi: 10.3390/insects16090939 (PMC12471267; doi:10.3390/insects16090939)
Supplement: Supplementary file 1 [file insects-16-00939-s001.zip › Table S1.pdf]

Table S1. The species and abundance of herbivorous insects collected under different grazing regimes.

Name	Sp+Su	Su+Au	Sp+Au	Annual	CK	Proportion（%）
Penthicus laelaps	1	5	0	8	7	1.72
Agrilus integerrimus	4	6	2	5	6	1.88
Phytoscaphus gossypii	21	0	0	0	10	2.53
Eodorcadion heros	1	1	0	1	0	0.25
Conocephalus chinensis	0	0	0	0	1	0.08
Lygaeus murinus	1	1	0	0	1	0.25
Maruca vitrata	0	0	0	0	1	0.08
Platyscelis hauseri	0	0	0	0	1	0.08
Anatolica nureti	0	0	0	2	1	0.25
Pyrrhocoris sibiricus	1	0	0	0	0	0.08
Potosia brevitarsis	8	10	2	8	13	3.35
Blaps femoralis	86	57	39	50	70	24.69
Microdera kraatzi alashanica	91	128	56	75	123	38.68
Maladera verticalis	5	2	0	6	2	1.23
Ampedus nigrinus	0	0	0	4	0	0.33
Anatolica ebenina	0	0	0	1	2	0.25
Bothynoderes punctiventris	0	1	0	0	2	0.25
Gryllotalpa unispina	3	5	2	5	5	1.64
Chrysochus chinensis	0	0	0	0	1	0.08
Dermestes dimidiatusab rosea	0	1	0	0	0	0.08
Trematodes tenebrioides	2	4	2	6	9	1.88
Maladera orientalis	0	0	0	1	0	0.08
Alydus zichyi	3	7	1	1	8	1.64
Brahmina faldermanni	31	15	14	9	79	12.10
Dorysthcnes paradoxus	4	1	0	3	1	0.74
Zichya tenggerensis	3	2	0	3	1	0.74
Chorthippus albonemus	2	6	1	2	2	1.06
Lygaeus equestris	2	2	1	0	0	0.41
Conorrhynchus pulverulentus	1	0	1	0	1	0.25
Chorthippus brunneus	1	0	0	2	0	0.25
Platyope mongolica	1	1	2	2	0	0.49
Eodorcadion multicarinatum	2	0	1	0	0	0.25
Celerio euphorbia Linnaeus	1	2	0	0	0	0.25
Eodorcadion virgatum	0	0	0	2	1	0.25
Asproparthenis secura	0	2	0	1	1	0.33
Mongolodectes alashanicus	0	0	0	0	1	0.08
Calliptamus abbreviatus	0	0	0	0	1	0.08
Penthicus alashanicus	0	1	0	1	0	0.16
Coriomeris nigridens	0	0	0	1	0	0.08
Mylabris calida	0	0	0	1	0	0.08
Mantichorula semenowi	0	1	0	2	0	0.25
Pseudocneorhinus sellatus	0	0	1	0	0	0.08

Bothynoderes declivis	0	0	0	2	0	0.16
Batocera rubus	0	0	0	1	0	0.08
Agrotis segetum	0	1	0	0	0	0.08
Anomala sulcipennis	0	2	0	0	0	0.16
Crambus pinellus	0	0	0	1	0	0.08
Chrysolina aeruginosa	0	0	0	1	0	0.08
Popillia quadriguttata	0	0	0	1	0	0.08
Total	275	264	125	208	351	100.00
