# Supplementary material for: Effect of Seasonal Grazing on Ground-Dwelling Insect Communities in the Desert Steppe of Ningxia
Source: Insects. 2025 Sep 6;16(9):939. doi: 10.3390/insects16090939 (PMC12471267; doi:10.3390/insects16090939)
Supplement: Supplementary file 1 [file insects-16-00939-s001.zip › Table S2.pdf]

Table S2. The species and abundance of predatory insects collected under different grazing regimes.

Name	Sp+Su	Su+Au	Sp+Au	Annual	CK	Proportion（%）
Harpalus sinicus	21	21	36	6	57	9.78
Harpalus pallidipennis	7	20	15	14	29	5.89
Camponotus herculeanus	1	5	0	0	3	0.62
Araneida3	4	0	3	0	2	0.62
Carabus brandti	0	2	0	1	0	0.21
Ommatius chinensis	3	2	2	0	2	0.62
Pterostichus gebleri	1	0	1	0	3	0.35
Araneida1	11	17	12	6	50	6.66
Araneida2	6	0	5	1	1	0.90
Cymindis binotata	0	0	0	0	1	0.07
Harpalus bungii	2	0	2	5	1	0.69
Hippodamia variegata	1	0	0	0	0	0.07
Corsyra fusula	4	5	6	0	8	1.60
Pseudotaphoxenus mongolicus	14	5	14	9	17	4.09
Pseudotaphoxenus brevipennis	0	2	2	1	1	0.42
Onthophagus sinicus	1	3	0	12	3	1.32
Cylindera elisae	51	27	5	5	23	7.70
Cataglyphis aenescens	71	277	66	48	137	41.54
Araneida3	4	0	5	5	4	1.25
Araneida4	3	1	1	0	7	0.83
Termites	31	34	0	2	34	7.00
Leiobunum species	14	11	24	4	1	3.74
Scolopendra	0	0	1	0	0	0.07
Labidura japonica	0	3	2	0	2	0.49
Scutigera coleoptrata	4	0	6	6	4	1.39
Calosoma chinense	2	0	0	0	0	0.14
Harpalus davidianus	1	0	2	0	0	0.21
Cicindela hybrida nitida	1	0	1	0	0	0.14
Araneida5	1	7	0	0	9	1.18
Araneida6	0	0	0	0	1	0.07
Dolichus halensis	0	0	0	1	0	0.07
Araneida7	0	2	0	0	0	0.14
Araneida8	0	0	2	0	0	0.14
Total	259	444	213	126	400	100.00
